# Dealing with dry waste disposal issues associated with ^177m^Lu impurities: a long-term challenge for nuclear medicine departments

**DOI:** 10.1186/s40658-023-00524-z

**Published:** 2023-01-09

**Authors:** Sylviane Prevot, Inna Dygaï-Cochet, Jean-Marc Riedinger, Jean-Marc Vrigneaud, Myriam Quermonne, Matthieu Gallet, Alexandre Cochet

**Affiliations:** 1 Service de Médecine Nucléaire, Centre G.F. Leclerc, 1 Rue Pr Marion, 21079 Dijon Cedex, France; 2 ImVIA, EA 7535, University of Burgundy, 9 Avenue Alain Savary, BP 47870, 21078 Dijon Cedex, France

**Keywords:** ^177^Lu-dotatate, Lutathera®, ^177m^Lu impurities, Storage, Waste

## Abstract

**Purpose:**

A strategy for management of radioactive waste associated with ^177^Lu-dotatate (Lutathera®) treatments was established in our institution, based on predicted storage times of 3–5 years extrapolated from the results of a 2-year measurement study. The aim of this work was to validate this strategy by identifying contaminants and confirming disposal based on the clearance level twice-the-background was within expected time frames.

**Methods:**

We conducted a prospective series of measurements of radioactive waste associated with the first 65 treatments administered. Sequential measurements of the first 45 vials used were performed on a dose calibrator to identify contaminants. Exposure rates in contact were monitored with a dose ratemeter on a 6-monthly basis for all waste stored: 46 empty vials, 19 vials partially used and 61 biohazard containers.

**Results:**

Initial median activity of the first vials used was 118 MBq [4–4188 MBq]. For each vial, the decay curve of activity obtained was adjusted to a bi-exponential model. The major component, representing 99.7% of the activity, has a median half-life of 6.6 days [5.7–7.2 days] corresponding to ^177^Lu. The second, representing only 0.3% of the activity and having a median half-life of 152 days [104–205 days] corresponding to ^177m^Lu, determines necessary storage times. Partially used vials can be disposed of after 5 years, other waste after 3 years. Compliance with the regulatory clearance level is achieved within expected time frames.

**Conclusion:**

Although only present as traces, ^177m^Lu associated with the direct production route results in major radioactive waste disposal issues for hospitals. Availability of radiopharmaceuticals without impurities appears to be crucial for an expanding use of targeted radionuclide therapy.

## Introduction

Council Directive 2013/59/Euratom of December 5, 2013 [1], re-emphasized the key role of optimization of protection, the ALARA concept, applying to all categories of exposure. Waste management strategies and environmental monitoring programs must be developed to ensure the best possible protection of the population. The license issued by national authorities to hold and make use of unsealed radioactive sources in nuclear medicine (NM) requires hospitals to control the radioactive waste that is generated. Prior analysis and measurements have to be performed each time a new radionuclide is intended to be used so that all practices can be optimized.

Lutetium-177 (^177^Lu) is very promising for use in targeted radionuclide therapy (TRT). According to recent results of the VISION trial on treatment of metastatic prostate cancer with ^177^Lu-PSMA-617 [2], a significant increase in the number of patients treated with ^177^Lu and, consequently, in the volume of radioactive waste produced is expected in the coming years. NM departments must therefore review and adapt their radiation risk control strategy to the characteristics and properties of this new marker. ^177^Lu decays to stable Hafnium-177 (^177^Hf) with a half-life of 6,64 days, emitting beta particles with maximum energy 497 keV (97%) together with low energy and low abundance gamma rays (208 keV, 11%; 113 keV, 13%), resulting in a low risk of external exposure when away from the source. Safe storage of waste can be organized in facilities designed for Iodine-131 high-energy gamma rays.

Assessing the impact of a growing use of ^177^Lu in our hospital, we focused on the concerns arising when starting treatments with ^177^Lu dotatate (Lutathera®, Advanced Accelerator Applications/Novartis), a somatostatin analogous peptide, indicated for the treatment of well-differentiated, metastatic gastro-entero-pancreatic neuroendocrine tumors [3]. On-site storage of waste was initially organized according to ^177^Lu half-life, for an expected time frame of 3 months corresponding to 13 half-lives. However, after 3 months of decay, exposure rates (ER) in contact with empty vials were still 14 times higher than the clearance level and they were no longer decreasing on a weekly basis. No waste could be disposed of. A study to determine optimum waste management procedures was therefore carried out. Necessary storage times ranging from 3 to 5 years were estimated from the results of a 2-year measurement study [4]. A waste management strategy was then established based on these time frames.

The purpose of this paper was to validate this strategy by:

- Identifying and quantifying long-lived contaminants.
- Confirming waste can be disposed of within predicted storage times.

## Material and methods

Single-dose Lutathera® vials contain a ready-to-use solution for infusion whose activity is 7.4 GBq (± 10%) at the date and time of administration. Recommended treatment regimen in adults consists of 4 infusions of 7.4 GBq each administered every 8 ± 1 weeks. A reduction in the administered activity up to 3.7 GBq may be justified for clinical reasons. In that case, a partially used vial with residual activity 3.7 GBq is discarded and stored for decay. Vials also contain metastable Lutetium (^177m^Lu) impurities, a ^177^Lu nuclear isomer associated with the direct production process [5]. 21.4% of ^177m^Lu decays to ^177^Lu with a half-life of 160.4 days via isomeric transition, 78.6% to ^177^Hf by beta emission [6].

Treatments are administered in a dedicated infusion room within the NM department, where dry waste is separated and collected in appropriate containers. ER in contact with all types of waste are measured at the end of every procedure. Non-radioactive waste is discharged. Radioactive waste is identified and transferred to dedicated storage for decay. Practical optimization of the volume of storage available was achieved by considering three separate types of waste: biohazard containers, empty (fully used) vials and partially used vials with residual solution of high activity.

The waste management strategy was established as follows:

- Empty vials and biohazard containers are held in storage for an estimated period of 3 years before disposal based on the French regulatory clearance level of twice-the-local-background (2*BKG = 0.1 µSv.h^−1^) in contact and total residual ^177m^Lu activity in vials kept below the exemption level of 1 MBq [1]. Vials are discarded in bags on a 6-monthly basis.
- Partially used vials are stored in their as-delivered lead container for 3 years, before being discarded in bags on a yearly basis. Bags are then stored for 2 additional years before expected disposal.

We conducted a prospective series of measurements of waste associated with the first 65 treatments, administered on an outpatient basis from November 2017 to July 2019. Data for Lutathera® vials used are presented in Table 1.

**Table 1:** Data for the 65 Lutathera® vials used for the first treatments administered

Vials	Treatments	Activity measured MBq (mean ± SD)		
	N	At reception time	At infusion time	Discarded post-infusion
Fully used (empty)	46	7562 ± 119	7404 ± 122	110 ± 14
Partially used	19		4118 ± 836	3800 ± 800

**Fig. 1:**
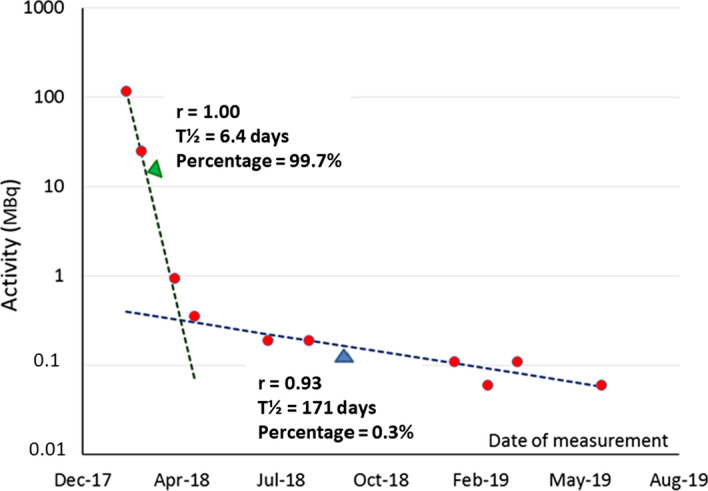
Plots of a bi-exponential decay with fitting curves and calculated parameters: half-life, percentage of contaminants, correlation coefficient (*r*)

Sequential measurements of the first 45 vials used were performed on a dose calibrator Veenstra Medi-404 (Medisystem, Guyancourt, FR) to identify and quantify contaminants. For each vial, the radioactive decay curve obtained was fitted to a bi-exponential model (Fig. 1). Initial activity of the longer-lived component was extrapolated from the second part of curves. These values were then subtracted from those measured in the first part to estimate the percentage of the fast-decaying contaminant.

ER in contact were monitored with a dose ratemeter identiFINDER 2 (High Tech Detection Systems, Massy, FR) on a 6-monthly basis for all types of waste stored: 61 biohazard containers, 46 empty vials and 19 partially used vials.

Waste disposal within predicted time frames allows validation of the strategy used.

## Results

Initial median activity was 118 MBq [4–4188 MBq]. Percentage and half-life of the two components identified are presented in Table 2. The major component, representing 99.7% [85.6–99.8%] of the activity, has a median half-life of 6.6 days [5.7–7.2 days] corresponding to ^177^Lu. The second, representing only 0.3% [0.2–1.7%] of the activity and having a median half-life of 152 days [104–205 days] corresponding to ^177m^Lu, determines necessary storage times.

**Table 2:** Data for radioactive decay curves obtained from sequential measurements of the 45 first vials used

	Vials	Percentage (%)	Correlation coefficient (r)	Half-life (Days)
Component	N	Median (range)	Median (range)	Median (range)
First	41	99.7 (85.6–99.8)	1.00 (0.96–1.00)	6.6 (5.7–7.2)
Second	45	0.3 (0.2–1.7)	1.00 (0.98–1.00)	152 (104–205)

ER measured in July 2021, in contact with all waste stored for decay since 2018, are presented in Tables 3, 4, and 5.

**Table 3:** Data for N empty vials and corresponding bags, measured in 2021 to determine (ER/2BKG) ratios

Period of treatments		First half 2018	Second half 2018	First half 2019
Vials July 2021	Number of vials stored (N)	19	9	18
	Storage time of vials (median [range] in months)	39.4 [36.9–42.3]	34.6 [31.3–36.5]	25.5 [23.4–29.2]
	(ER/2BKG) Ratio* (median [range])	0.5 [0.5–0.6]	0.7 [0.6–0.9]	1.1 [0.8–1.5]
Bags July 2021	Number of bags containing N vials	1	1	1
	(ER/2BKG) Ratio*	1.0	2.1	4.5
	Additional storage time required (months)	0	6	12
	Calculated date of disposal	July 2021	December 2021	July 2022
	Compliance with predicted storage times (3 years)	Yes	Yes	Yes

*ER  Exposure rate measured in contact with the container; 2BKG = 0.1 μSv.h^−1^ regulatory clearance level of twice-the-background

**Table 4:** Data for *N* biohazard containers measured in July 2021 to determine (ER/2BKG) ratios

Period of treatments	November 2017–June 2018	Second half 2018	First half 2019
Number of biohazard containers stored (N)	18	17	26
Storage time (median [range] in months)	40.1 [37.5–44.8]	34.7 [31.9–37.1]	26.7 [23.1–30.9]
(ER/2BKG) Ratio*—July 2021(median [range])	0.7 [0.5–1.2]	0.7 [0.6–3.0]	1.1 [0.7–2.3]
Additional storage time required (months)	0	12	6
Calculated date of disposal	July 2021	July 2022	December 2021
Compliance with predicted storage times (3 years)	Yes	Yes (94.1%)**	Yes

*ER   Exposure rate measured in contact with the container; 2BKG = 0.1 μSv.h^−1^ regulatory clearance level of twice-the-background

**Only 1 of 17 containers with an ER of 0.3 μSv.h^−1^ requires 12 additional months for decay. Others were disposed of in July 2021

**Table 5:** Data for N partially used vials discarded in 2 bags, measured to determine (ER/2BKG) ratios

Period of treatments		December 2017–December 2018	First half 2019
Vials July 2021	Number of vials stored (N)	10	9
	Storage time of vials (median [range] in months)	34.6 [32.2–43.6]	29.1 [26.1–30.9]
	Residual activity per vial (median [range] in kBq)	100 [26–165]	210 [105–615]
	(ER/2BKG) Ratio* (median [range])	8 [4.0–14]	15.0 [8.3–41.3]
	Additional storage time required (months)	24	36
	Calculated date of disposal	July 2023	July 2024
	Compliance with predicted storage times (5 years)	YES	YES
Bags July 2021	Number of bags containing N vials	1	1
	Total residual activity in one bag (MBq)	0.92	2.32
	(ER/2BKG) Ratio*	50	183
	Additional storage time required (months)	36	48
	Calculated date of disposal	July 2024	July 2025
	Compliance with predicted storage times (5 years)	NO	NO

*ER  Exposure rate measured in contact with the container; 2BKG = 0.1 μSv.h^−1^regulatory clearance level of twice-the-background

*Empty vials *(Table 3)*:* ER of each vial from the first semester 2018 was the local BKG (0.05 µSv.h^−1^). In contact with the bag of 19 vials, ER was complying with the clearance level 2BKG. This bag was disposed of.

An additional 6 months of storage were necessary for vials of the second half of 2018, 12 months for those of the first semester 2019.

*Biohazard containers *(Table 4)*:* 97.1% of waste collected in 2018 were disposed of. Only one container of the second half of 2018, with an ER of 0.3 µSv.h^−1^ (6*BKG), required one additional year of storage.

Ten of the 26 containers of the first semester 2019 (38.5%) were disposed of after 2 years of decay. Others were maintained in storage for 6 additional months.

*Partially used vials *(Table 5)*:* After a median storage time of 34.6 months, median activity of the 10 vials used in 2018 was 100 kBq [26–165 kBq]. ER were still 4–14 times higher than the clearance level, requiring 2 additional years of decay. When discarded in a bag, total residual activity was about 1 MBq and ER in contact with the bag was 50 times higher than the clearance level. Three additional years of storage were necessary before the bag of 10 vials could be disposed of instead of 2 years if vials were disposed of on an individual basis.

Vials used in 2019 had a median activity of 210 kBq [105–615 kBq], and their ER was 8–41 times higher than the clearance level: 3 additional years of decay were required. ER in contact with the bag of 9 vials confirmed 4 additional years of storage would be necessary before disposal.

## Discussion and conclusion

This study demonstrated that although only present as traces, ^177m^Lu determines minimum storage times, below which dry waste cannot be disposed of without activating the alarm of the portal monitor used for radiological output checks of containers. Long-term storage must therefore be organized according to ^177m^Lu half-life of 160.4 days within appropriate premises. Five years of decay are necessary before disposal of individual vials with residual solution, 3 years for empty vials and biohazard containers. The waste management strategy established ensures that compliance with the regulatory clearance level is achieved within predicted storage times.

In our institution, the volume of storage available had to be doubled since the beginning of Lutathera® therapy. Additionally, a cold room, dedicated to the storage of long-lived putrescible and biohazardous waste, was built to optimize the protection of staff exposed to infectious hazard by avoiding extreme heat in the facility.

Assessing the impact of a growing use of ^177^Lu in hospitals, waste management strategies to be developed will depend on the production process used by suppliers. ^177m^Lu associated with the direct production route results in major waste disposal issues for NM departments. Primary emphasis must be placed on minimization of the volume of long-lived waste to ensure and maintain compliance with regulatory requirements. Availability of radiopharmaceuticals without impurities appears to be crucial for an expanding use of TRT.

## Data Availability

Manuscript.
